# Resource Allocation in Uplink NOMA-IoT Based UAV for URLLC Applications

**DOI:** 10.3390/s22041566

**Published:** 2022-02-17

**Authors:** Rana Karem, Mehaseb Ahmed, Fatma Newagy

**Affiliations:** 1 Department of Electronics and Communications Engineering, Misr International University (MIU), Cairo 11828, Egypt; mehasb.ahmed@miuegypt.edu.eg; 2 Department of Electronics and Communications Engineering, Ain Shams University (ASU), Cairo 11517, Egypt; fatma_newagy@eng.asu.edu.eg

**Keywords:** internet of things, non-orthogonal multiple access, resource allocation, ultra reliable low latency communication, unmanned aerial vehicles, uplink transmission

## Abstract

One of the main targets of future 5G cellular networks is enlarging the Internet of Things (IoT) devices’ connectivity while facing the challenging requirements of the available bandwidth, power and the restricted delay limits. Unmanned aerial vehicles (UAVs) have been recently used as aerial base stations (BSs) to empower the line of sight (LoS), throughput and coverage of wireless networks. Moreover, non-orthogonal multiple access (NOMA) has become a bright multiple access technology. In this paper, NOMA is combined with UAV for establishing a high-capacity IoT uplink multi-application network, where the resource allocation problem is formulated with the objective of maximizing the system throughput while minimizing the delay of IoT applications. Moreover, power allocation was investigated to achieve fairness between users. The results show the superiority of the proposed algorithm, which achieves 31.8% delay improvement, 99.7% reliability increase and 50.8% fairness enhancement when compared to the maximum channel quality indicator (max CQI) algorithm in addition to preserving the system sum rate, spectral efficiency and complexity. Consequently, the proposed algorithm can be efficiently used in ultra-reliable low-latency communication (URLLC).

## 1. Introduction

Due to their mobility and flexibility, unmanned aerial vehicles (UAVs)—also called drones—have become popular and have a wide range of applications. These applications include—though are not confined to—monitoring the environment, fertilizing and dusting crops, searching for mines, police monitoring, and operations of rescue. UAVs have received great attention thanks to its lower cost, high mobility, wide coverage and ease of deployment and flexibility [1]. These applications are not restricted to industrial and civilian fields only, but can also be extended to military applications [2].

In the near future, UAVs will be highly deployed in many applications in wireless networks, especially 5G networks, to serve ultra-reliable low-latency communication (URLLC) [3]. UAVs can present either in a single-UAV or multi-UAV system [4]. UAVs can be utilized as airborne base stations and flying relays to provide LoS connections to improve the coverage and power consumption for IoT devices. The recent era will witness billions of connections of Internet of Things (IoT) devices that will be served simultaneously [5]. Smart mobiles, vehicles, home appliances, and sensors are examples of these IoT devices [6].

The fifth generation (5G) network is intended to provide coverage for high-density devices with various applications of diverse quality of service (QoS) requirements [7]. Non-orthogonal multiple access (NOMA) is a promising multiple access technology which can face the challenging requirements of high throughput and low latency accompanied with the traffic of IoT devices. The basic concept of NOMA lies in the ability of NOMA to serve numerous users simultaneously over the same resource block (RB) to increase the spectrum efficiency [8]. However, due to simultaneous transmission, interference occurs between users sharing the same RB, so that successive interference cancellation (SIC) is used to detect the signal [9]. The exploitation of UAVs combined with NOMA is used to support the required massive connections of IoT devices and also provide long transmission range for IoT devices with limited transmission power capabilities. UAVs can easily overpass the IoT nodes, collect the information data and then transmit them to the data center or the other IoT devices [4]. IoT is accommodating numerous various applications with extremely precise performance requirements. Delay is one of the essential key metrics and one of the biggest challenges facing IoT applications [10]. In this paper, the UAV as a base station was exploited with NOMA to support URLLC IoT network with devices of different applications; each application has its own traffic parameters and delay requirements. A resource and power allocation scheme was proposed to manage the different QoS requirements for each application in uplink networks achieving high spectral efficiency, fairness and data rate as well as improving the delay and reliability.

The main contributions of this research are listed as follows:

- The uplink NOMA-based transmission system utilizing the UAV as a base station is modeled. Then, the resource allocation problem is formulated with the aim of maximizing the sum rate, taking into consideration the different delay requirements to serve URLLC applications.
- The proposed scheduler allocates resources jointly in both time domain and frequency domain based on the IoT devices parameters. Delay limits and priority are used by time domain; then the buffer status report (BSR) and channel quality indicator (CQI) control the frequency domain scheduler decision to allocate resources. In addition, a power allocation scheme is proposed to achieve fairness between the users allocated the same RB regardless of the different channel conditions.
- Unlike the previous works, the novelty of the presented algorithm lies in its ability to consider both the strict delay requirements of IoT devices and the system throughput while ensuring high reliability and fairness, where simulations are performed to evaluate the proposed scheduling algorithm performance. The results demonstrate the effectiveness of the proposed algorithm to serve URLLC traffic with restricted delay limits, due to the significant enhancement in delay, reliability and fairness, in addition to maximizing the sum data rate and spectral efficiency while achieving the same system complexity when compared to the maximum channel quality indicator (max CQI) algorithm.

## 2. Related Works

This section presents the state-of-the-art in resources scheduling in uplink 5G networks, UAV communication, and NOMA-based URLLC systems. Resource allocation and scheduling techniques in uplink 5G networks have been studied in many research papers presented in this literature. In [8], the authors proposed two resource allocation algorithms; the first one is the local rate maximization (LRM) in which the subcarrier is allocated to the user which gives the maximum rate on the chosen subcarrier. The second approach is global objective maximization (GOM) in which the allocated subcarrier is that which achieves maximum increase in the objective function.

A weighted sum rate maximization problem is modeled in [11], then a subcarrier allocation scheme which is based on iterative water-filling (IWF) algorithm is introduced. Its main idea is to initially begin with all devices allocated to all subchannels then remove the subchannel-device pair which gives the worst gain and power; repeatedly doing so until it meets the constraint that *L* devices are allocated to each subchannel [11]. The authors in [12] came up with a many-to-many matching model, where each subchannel forms a preference list based on the system throughput and each user creates its own preference list based on the received power. Iterative addition or substitution processes for the users to the subchannels are performed; for the purpose of enhancing the system performance until there is no more enhancement [12]. Reference [13] shows a two-sided matching and swapping technique, where firstly, each device forms its preference list based on channel gain and data rate, then each subchannel receives requests from the demanding devices and chooses the *L* devices with the highest energy efficiency. Then swapping operations are performed if and only if it is accompanied by an enhancement in energy efficiency [13]. UAV cellular networks were investigated with 5G technologies in [14,15] considering the channel model but the resource allocation problem was not inspected. In [16], backscatter communication technology, which is based on reflecting the incident wireless signals for the purpose of data collection, is investigated. The mobility of UAVs is exploited for maximizing the energy efficiency while optimizing the backscatter devices allocation and UAV trajectory [16]. The authors in [17] proposed NOMA scheme with index modulation to reduce the effect of contention, interference and collision in grant-free access. However, the system throughput was not considered. The authors in [18] used resource slicing and presented two user clustering mechanisms to meet the delay constraints of time stringent applications in uplink NOMA. NOMA technology is used in [19] and the resources are classified into shared and private. If the transmission and delay requirements of users cannot be achieved by the shared resources, then the private resources can be used [19]. Unlike the studies, the proposed algorithm is the first to consider the restricted delay limits of the IoT devices in addition to throughput maximization which makes the proposed algorithm suitable for URLLC applications.

The following sections in the rest of the paper are organized as follows. In Section 3, the system model is illustrated for the uplink of IoT devices served by UAV using NOMA access technique. Section 4 shows the problem formulation with the purpose of delay minimization and maximizing the network sum rate. Section 5 demonstrates the delay-rate optimization scheduling algorithm and the fairness optimization power allocation algorithm. Section 6 shows the performance evaluation of the proposed algorithm through the simulation results. Finally, the conclusion of the presented work is discussed in Section 7.

## 3. System Model

**Figure 1:**
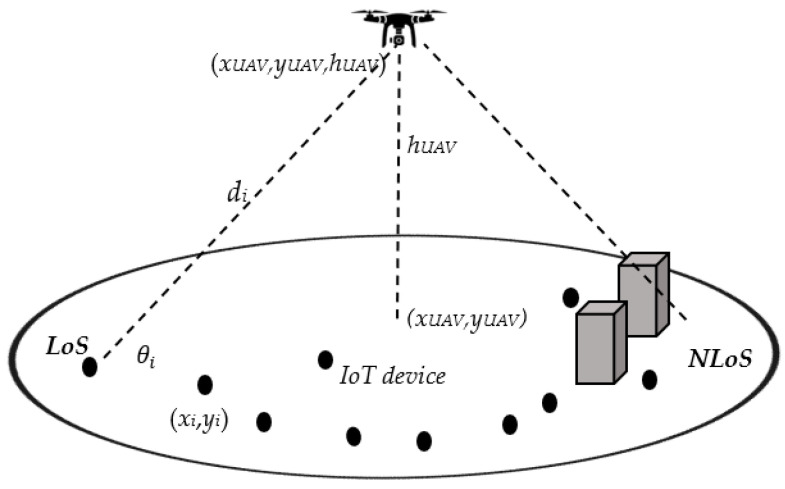
Illustration of the elevation angle in case of static UAV [21].

As shown in Figure 1, a UAV-assisted IoT network is considered where the UAV serves as an aerial base station, all IoT devices are served by a UAV that covers a cell with radius $R_{c}$. The UAV is located at 3D coordinates (*x_UAV_*, *y_UAV_*, *h_UAV_*); assume that ($x_{UAV}=0$, $y_{UAV}=0$, $h_{UAV}$); i.e., the UAV is at the center of the cell with altitude *h_UAV_*; such that (*h_min_* ≤ *h_UAV_* ≤ *h_max_*) where *h_min_* and *h_max_* are the minimum and maximum allowable heights of the UAV. Assume that there are *N* ground IoT devices; each device is equipped with a single antenna. These devices are distributed randomly in the cell covered by the UAV; each device is located at (*x_i_*, *y_i_*) where *i* = {1, 2, …, *N*}. Each IoT device has a packet arrival rate $\lambda_{i}$ and transmits minimum rate *R_i_* with delay limit *D_i_*. Each IoT device has a transmitted power *P_i_* (*P_min,i_* ≤ *P_i_* ≤ *P_max,i_*) where *P_min,i_* and *P_max,i_* are the minimum and maximum transmitted power of IoT device *i* respectively. Path loss between IoT device *i* to the UAV expresses the large-scale fading component which is given by

(1) $$PL_{i}=\frac{A}{1+ae^{-b\;\left(\theta_{i}-a\right)}}+B_{i},$$

(2) $$A=\eta_{LOS\;}-\eta_{NLOS},$$

(3) $$\;B_{i}=20\;log_{10}\left(d_{i}\right)+20\;log_{10}\left(\frac{4\pi f_{c}}{c}\right)+\eta_{NLOS},$$

where $f_{c}$ is the carrier frequency, c is the light speed, $\eta_{LOS}$, $\eta_{NLOS}$, a, b are constants related to the propagation environment either urban, suburban, dense urban or high-rise urban environments. Considering air to ground (A2G) communication between the UAV and the ground IoT devices, thus, each device can have line of sight (*LoS*) view or non-line of sight (*NLoS*) view with respect to the aerial base station with certain probability. *NLoS* occurs when the propagation path is partially or fully obscured by physical obstacles. The *LoS* probability or *NLoS* probability are highly dependent on the device location, environment and the elevation angle between the device and the UAV, as illustrated in Figure 1. $Pr_{LoS_{i}}$, $Pr_{NLoS_{i}}$ are the probabilities of IoT device *i* having *LoS* link or having non-line of sight (*NLoS*) link respectively between the UAV and IoT device, which could be calculated as [20]

(4) $$Pr_{LoS_{i}}=\frac{1}{1+aexp\left(-b\left[\theta_{i}-a\right]\right)}\;,\;$$

(5) $$Pr_{NLoS_{i}}=1-Pr_{LoS_{i}}$$

where $\theta_{i}$ is the elevation angle (measured in “degree”) between IoT device *i* and the *UAV* as illustrated in Figure 1; it can be calculated as

(6) $$\theta_{i}=\frac{180}{\pi }\sin^{-1}\left(\frac{h_{UAV}}{d_{i}}\right)$$

$d_{i}$ is the distance between the IoT device *i* at (*x_i_*, *y_i_*) and the *UAV* located at ($x_{UAV}$, $y_{UAV}$, $h_{UAV}$) is

(7) $$d_{i}=\sqrt{{\left(x_{UAV}-x_{i}\right)}^{2}+{\left(y_{UAV}-y_{i}\right)}^{2}+{\left(h_{UAV}\right)}^{2}}$$

The *UAV* uses the NOMA access technique to communicate with the IoT devices. Assume that the total bandwidth allocated to the *UAV* is *B*, which is divided equally into *K* orthogonal subcarriers. Let *b_k,i_*(*t*) is the subcarriers assignment index, where *b_k,i_*(*t*) = 1 means that subcarrier *k* is allocated to device *i*, otherwise *b_k,i_*(*t*) = 0. There are maximum *L* IoT devices allowed to be scheduled over a single subcarrier at the same time and each IoT device gets exactly one subcarrier for simplicity. IoT devices can give preferences for the resource blocks, and their preferences are considered based on their channel quality indicator (CQI) [22] which depends on the channel quality between the *UAV* and IoT devices. The signal to noise plus interference ratio (SNIR) of IoT device *i* on subcarrier *k* is modeled as

(8) $$\Gamma_{k,i}\left(t\right)=\frac{b_{k,i}\left(t\right)p_{k,i}\left(t\right){\left|g_{k,i}\left(t\right)\right|}^{2}}{\sum_{f=1,\;{\left|g_{k,f}\left(t\right)\right|}^{2}<{\left|g_{k,i}\left(t\right)\right|}^{2}}^{N}b_{k,f}\left(t\right)p_{k,f}\left(t\right){\left|g_{k,f}\left(t\right)\right|}^{2}+\sigma^{2}},$$

$g_{k,i}\left(t\right)$ is the channel gain between the *UAV* and IoT device *i* on subcarrier *k*. $g_{k,i}\left(t\right)$ which is invariant in one time slot but varies over different time slots, can be modeled as:

(9) $$g_{k,i}\left(t\right)=\sqrt{PL_{k,i}}h_{k,i}\left(t\right),$$

where $h_{k,i}\left(t\right)$ is the small scale fading of complex Gaussian distribution given by $h_{k,i}\left(t\right)$*∼ CN*(0,$\sigma^{2}$). URLLC uses short packets to ensure low latency transmission, so Shannon capacity, which acts as the upper bound in terms of data rate, can no longer be applied. Consequently, the user data rate at finite blocklength transmission [23] is given by

(10) $$R_{k,i}\left(t\right)=\log_{2}\left(1+\Gamma_{k,i}\left(t\right)\right)-\sqrt{\frac{V_{k,i}}{m}\;}\;Q^{-1}\left(\varepsilon \right)$$

where, the decoding error probability ε [24] is given by

(11) $$\varepsilon =Q\left(\ln2\sqrt{\frac{m}{V}}\;\left(\log_{2}\left(1+\Gamma \right)-\frac{D}{m}\right)\right),$$

Such that the Q function and V are defined respectively as

(12) $$Q\left(x\right)=\frac{1}{\sqrt{2\pi }}\int_{x}^{\infty }e^{\frac{-t^{2}}{2}}\;dt$$

(13) $$V_{k,i}=1-{\left(1+\Gamma_{k,i}\right)}^{-2}\;$$

where D states the packet size, and m represents the blocklength of the channel.

The sum rate can be calculated as

(14) $$\;R_{sum}=\overset{K}{\underset{k=1}{\sum }}\sum_{i=1}^{N}R_{k,i}\left(t\right),$$

**Figure 2:**
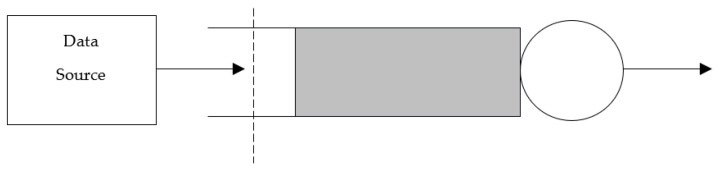
Queuing system model of the IoT device [26].

Assume that packet arrival of the IoT device *i* follows a Poisson process with the average arrival rate as $\lambda_{i}$ packets per second. The average service rate of the same IoT device is assumed to be *μ_i_* packets per second. Both $\lambda_{i}$ and *μ_i_* are statistically identical and independent distributed. The queuing system model of the IoT device is shown in Figure 2. Then, the average delay $\;d_{av,i}$, which is based on the *M/M/1* queuing model [25], can be given by the following formula:

(15) $$d_{av,i}=\frac{S_{i}}{R_{av,i}-S_{i}\lambda_{i}}$$

where *S_i_* and $R_{av,i}$ are the packet size and the average rate of IoT device *i* respectively.

$R_{av,i}$ can be given as the rate of device *i* averaged over time as follows:

(16) $$R_{av,i}=\frac{1}{T}\overset{T_{slots}\;}{\underset{t=1}{\sum }}\overset{K}{\underset{k=1}{\sum }}R_{k,i}\left(t\right)b_{k,i}\left(t\right),$$

where $T_{slots}$ represents the total number of time slots.

## 4. Problem Formulation

The main objective is to maximize the sum data rate of all users while achieving minimum device delay through selecting the assignment index $b_{k,i\;}$ at every time slot. Hence, the resource allocation problem is formulated with data rate maximization objective function as follows:

(17) $$\underset{\left\{b_{k,i}\left(t\right)\right\}}{\mathrm{Max}}\overset{K}{\underset{k=1}{\sum }}\sum_{i=1}^{N}R_{k,i}\left(t\right)$$

(18) $$s.t.\;C1:\overset{K}{\underset{k=1}{\sum }}b_{k,i}\left(t\right)R_{k,i}\left(t\right)\;\geq \;R_{min,i}$$

(19) $$C2:\overset{K}{\underset{k=1}{\sum }}b_{k,i}\left(t\right)p_{k,i}\left(t\right)\;\leq \;P_{max}$$

(20) $$C3:\;b_{k,i}\left(t\right)\;\in \left\{0,1\right\}$$

(21) $$C4:\;\overset{K}{\underset{k=1}{\sum }}b_{k,i}\left(t\right)p_{k,i}\left(t\right)\;\geq \;0$$

(22) $$C5:\overset{N}{\underset{i=1}{\sum }}b_{k,i}\left(t\right)\;\leq \;L$$

(23) $$C6:\;d_{av,i}<D_{i}$$

(24) $$C7:\;h_{min}<h_{UAV}<h_{max}$$

where *P_max_* is the maximum available transmission power of the IoT device. *C*1 is the minimum required data rate for all users to ensure QoS. *C*2 means that any IoT device transmit power cannot exceed *P_max_*. *C*3 and *C*4 are the constraints of the assignment matrix *b_k,i_* and power *p_k,i_*. *C*5 mentions that one subcarrier cannot be allocated to more than *L* users at the same time. *C*6 is the delay constraint, states that the average delay of the device *i* ($d_{av,i}$) should not exceed the delay limit requirement *D_i_*. *C*7 is the UAV height constraint between the minimum and maximum allowable altitudes.

The optimization problem is non-convex due to the coupled variables $b_{k,i}$ and $p_{k,i}$ in (20) and (21). Hence, the proposed algorithm uses a linear weighted utility function to overcome the complexity of the optimization problem (17).

## 5. Proposed Algorithm

**Figure 3:**
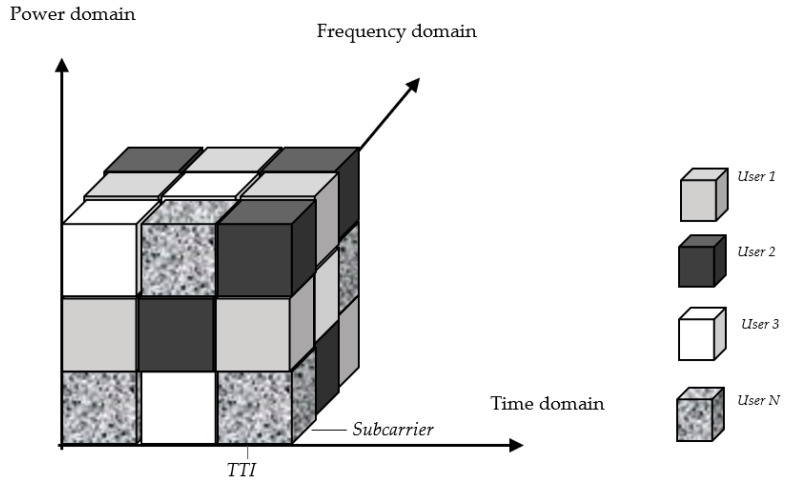
General NOMA resource allocation.

**Figure 4:**
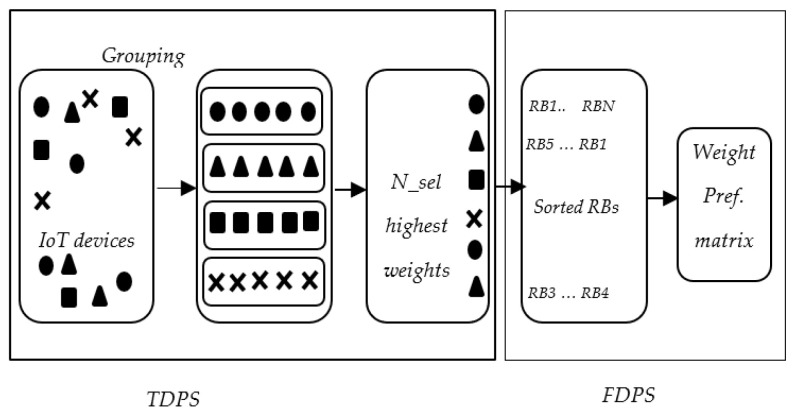
Illustration of the proposed scheduler.

The optimization problem is non-convex due to the coupled variables $b_{k,i}$ and $p_{k,i}$ in (20) and (21). To find a simplified solution, the proposed scheduler in this section exploits the convexity property of the sub-problems [27] for the main non-convex problem (17). In addition, the proposed algorithm makes use of the resource element structure in NOMA which takes one subcarrier in frequency and one time symbol known as transmission time interval (TTI) [28], as demonstrated in Figure 3. Hence, the proposed algorithm operates in two domains, as illustrated in Figure 4. The first is delay minimization to guarantee the delay requirement of each IoT device which is performed by a time domain packet scheduler (TDPS). The second domain is rate maximization to increase the spectral efficiency and maximize the system sum rate which is performed by a frequency domain packet scheduler (FDPS).

The optimum height of the UAV should be calculated to solve constraint C7. The optimal UAV elevation angle $\theta_{optimum}$ was derived for each environment in [29] to cover a circular cell of certain radius in A2G communication independent of the multiple access technique used. Thus, according to [30]; the values of $\theta_{optimum}$ given by (25) for UAV placement corresponding to each environment either suburban, urban, dense urban or high-rise urban environments are illustrated in Table 1. Thus, the optimum UAV altitude $h_{optimum}$ to cover a cell of radius $R_{c}$ can be given by

(25) $$\;\theta_{optimum}=\tan^{-1}\left(\frac{h_{optimum}}{R_{c}}\right)$$

### 5.1. Time Domain Packet Scheduling (TDPS)

The main function of TDPS is to select a certain number of IoT devices to be scheduled in the next TTI; this process should guarantee that there are no devices exceed their maximum delay limits by minimizing the average delay. Hence, the optimization problem for time domain scheduling can be formulated using (15) as

(26) $$\min\;d_{av,i}=\frac{S_{i}}{R_{av,i}-S_{i}\lambda_{i}},$$

The device selection criteria in TDPS is based on a weighted delay metric which is essentially needed to prioritize the devices requests. The metric function is based on delay [31] to satisfy the delay constraint C6. The UAV uses the information in the BSR to compute the metric function for each requesting device. This metric aims to order the IoT devices requests to control the TDPS decision. The metric considers average delay and buffer status reports [32] as follows:

(27) $$M_{i}=\alpha_{j}\;w_{i,j}d_{av,i}$$

where $\alpha_{j}$ states the priority of each traffic group, where the TDPS categorizes stations according to their delay limits into *j* groups, and $w_{i,j}$ is the traffic weight of user *i* in traffic class *j*, this weight can be calculated as

(28) $$\;w_{i,j}=\frac{q_{i,j}}{bl},$$

where $q_{i,j}$ is the queue size of user *i* in traffic group *j*, and *bl* is the buffer length to avoid buffer overflow of the IoT device.

After grouping and calculating metric for the IoT devices that send scheduling requests. The TDPS selects maximum *N_max_* = *KL* devices to be scheduled in the next TTI, since there are *L* devices can be scheduled over the same resource block according to constraint C5 (22). Consider *N_sel* to be the number of chosen IoT devices to be scheduled. The rejected users send scheduling requests in the next TTI.

### 5.2. Frequency Domain Packet Scheduling (FDPS)

The main purpose of FDPS is allocating resources for IoT devices based on channel conditions (CQI) to increase spectral efficiency [32]. FDPS allocates resources to the IoT devices chosen by TDPS. Thus, the optimization problem of FDPS could be formulated as

(29) $$\underset{\left\{b_{k,l}\left(t\right)\right\}}{\mathrm{Max}}\overset{K}{\underset{k=1}{\sum }}\sum_{l=1}^{N\_sel}R_{k,l}\left(t\right)$$

(30) $$s.t\;C1:\sum_{k=1}^{K}b_{k,l}\left(t\right)R_{k,l}\left(t\right)\;\geq \;R_{min,l}$$

(31) $$C3:\;b_{k,l}\left(t\right)\;\in \left\{0,1\right\}$$

(32) $$C5:\overset{N\_sel}{\underset{l=1}{\sum }}b_{k,l}\left(t\right)\;\leq \;L$$

In the proposed algorithm in FDPS each of the selected users prioritizes the RBs, where each IoT device sorts the RBs in descending order of CQI. The CQI value is an indication for the channel quality of each user on each subchannel. CQI value ranges from 0 to 15. The higher the value, the better the channel quality and vice versa. The CQI value is determined according to the estimated value of SINR [33]. Therefore, a preference matrix is formed containing the CQI values of all users on all RBs such that $\;CQI_{i,k}$ is the CQI value of user *i* on RB *k*; where the rows correspond to the IoT devices and the columns are related to the RBs. Each IoT device’s preferences (CQI values) are listed in a row in this matrix; such that a higher CQI value indicates that the IoT device prefers this RB.

A weighted preference matrix is then formed which is based on the weighted metrics (27) computed in TDPS and the preferences determined in FDPS, and can be given by

(33) $$\;f_{obj}=Diag\left(M_{1xN\_sel\;}\right)\;CQI_{N\_sel\;xK}\;$$

where $M_{1xN\_sel\;}$ is a row vector of the metrics of the selected devices and $\;CQI_{N\_sel\;xK}\;$ is the preference matrix which contains the CQI values of the selected devices on all RBs. Hence, the problem in (29) can be easily modified to

(34) $$\underset{\left\{b_{k,l}\left(t\right)\right\}}{\mathrm{Max}}-Diag\left(M_{1xN_{sel}}\right)\;CQI_{N_{sel}xK}b_{k,l}\left(t\right)$$

(35) $$s.t\;C3:\;b_{k,l}\left(t\right)\;\in \left\{0,1\right\}$$

(36) $$C5:\;\overset{N\_sel}{\underset{l=1}{\sum }}b_{k,l}\left(t\right)\;\leq \;L$$

The problem in (34) can be solved using binary integer programming which is used to solve the constrained problem to maximize $\;f_{obj}$ The algorithm (Algorithm 1) to find the optimum allocation matrix is listed in detail as shown in the pseudo code.
**Algorithm 1.** Joint Delay-Rate Optimization Scheduler **Input:**
*T*, *K*, *N*, λ, $P_{min}\;\&\;P_{max}$, *j*, *L* **Output:**
$\left\{s\right\}b_{k,i}$  1:  Initialize: N_max_ = L * K  2:  **Step 1: chooses the *N_max_* devices with the highest metrics in TDPS.**  3:  Arrange IoT devices in *j* groups according to the application type.  4:  **For**
*T_s_* = 1 to *T_s_* = *T*
**do**  5:     *R* = IoT devices send scheduling requests  6:     **If**
*R* =< *N_max_* go to Label  7:     **else**, **do**  8:        **For**
*i* = 1 to *i* = *R*
**do**  9:        Compute the weight of device *i* (27):$M_{i}=\alpha_{i}\;w_{i,j}d_{av,i}$10:  **End for**11:     Arrange IoT devices of each group in descending order of the weight ($M_{i}$)12:  Choose the *N_max* nodes which have the maximum weight to be scheduled in this TTI.13:  Label:      Choose the R nodes to be scheduled in this TTI.14:     **End If**15:     *N_sel* = the selected nodes to be scheduled16: **Step 2: assign each IoT device a resource block**17:  **For**
*i* = 1 to *i* = *N_sel*
**do**18:  Form the preference matrix for each device *i* to all available RBs based on the CQI value.19:  Form the objective function which is the weighted preference matrix $f_{obj}$.20:  Initialize $\hat{k}$ = 1, correspondingly set $f_{max}=f_{obj}$.21:  **if** a solution $\overset{`}{k}\epsilon \left\{\;k\epsilon K|\;f_{obj}\rangle f_{max}\right\}$ can be found: update $\hat{k}$ = $\overset{`}{k}$, and $f_{max}=f_{obj}$.22:  **End for**23:  Delay of the scheduled nodes is cleared, but that of waiting nodes is incremented.24:  Rejected nodes send scheduling requests in the next TTI.25: **End For**

In the Joint Delay-Rate Optimization Scheduler, the UAV receives scheduling requests in every TTI. In TDPS, the UAV classifies the requests to groups according to the application. Then, the metric function is computed for all requesting devices using (27). The scheduler selects *N_sel* devices with the highest metrics. Then, in FDPS, the RBs allocated to each IoT device are chosen with the objective to maximize the weighted preference matrix in (33).

### 5.3. Power Allocation Algorithm

The Uplink power allocation scheme is used to ensure fair data rates between users sharing the same RB [34]. Consider two users share the same RB, if $g_{k,n}$ > $g_{k,f}$ then according to (8) the SNIR of near and far users respectively are given as

(37) $$\;\Gamma_{k,n}\left(t\right)=\frac{p_{k,n}\left(t\right){\left|g_{k,n}\left(t\right)\right|}^{2}}{p_{k,f}\left(t\right){\left|g_{k,f}\left(t\right)\right|}^{2}+\sigma^{2}}$$

(38) $$\Gamma_{k,f}\left(t\right)=\frac{p_{k,f}\left(t\right){\left|g_{k,f}\left(t\right)\right|}^{2}}{\sigma^{2}},$$

Assuming that the UAV will decode the signal of the nearest user first then decode the signal of the far user using SIC. To achieve fair data rates for both users, then,

(39) $$\frac{p_{k,n}\left(t\right){\left|g_{k,n}\left(t\right)\right|}^{2}}{p_{k,f}\left(t\right){\left|g_{k,f}\left(t\right)\right|}^{2}+\sigma^{2}}\;\frac{p_{k,f}\left(t\right){\left|g_{k,f}\left(t\right)\right|}^{2}}{\sigma^{2}},$$

Let ${\left|g_{k,n}\left(t\right)\right|}^{2}=g_{N}$ as the gain of the near user and ${\left|g_{k,f}\left(t\right)\right|}^{2}=g_{F}$ as the gain of the far user. Thus, the power allocation coefficients $a_{N}$ and $a_{F}$ of the near and the far users respectively can be derived as

(40) $$\frac{a_{N}P_{max}g_{N}}{a_{F}Pg_{F}+\sigma^{2}}\;=\;\frac{a_{F}P_{max}g_{F}}{\sigma^{2}},$$

Let $\rho =P_{max}/\sigma^{2}$, then divide both sides by $\sigma^{2}$

(41) $$\frac{a_{N}\rho g_{N}}{a_{F}\rho g_{F}+1}\;=\;\frac{a_{F}\rho g_{F}}{1},$$

(42) $$g_{F}^{2}\;\rho^{2}a_{F\;}^{2}+(g_{F}+g_{N})\rho a_{F\;}-\rho g_{N\;}=0$$

(43) $$a_{F\;}=\frac{-\left(g_{N}+g_{F}\right)\pm \sqrt{{\left(g_{N}+g_{F}\right)}^{2}+4\rho g_{F}^{2}g_{N}}}{2g_{F}^{2}\;\rho }$$

where 0 <$\;a_{N}\;$< 0.5 because $a_{F}\;$>$\;a_{N}$ and $a_{N}+a_{F}=1$.

## 6. Simulation Results

In this section, simulation is introduced to evaluate the proposed algorithm performance. Consider a single UAV covering a cell of radius $R_{c}\;$= 1 km; assuming a suburban environment. An aerial UAV is centered and placed at the optimum height of the cell according to the values in Table 1. Consider IoT devices (N = 300) randomly deployed in the cell, IoT devices are supporting different applications, assuming there are 4 groups of IoT devices each has its unique traffic parameters. The maximum transmit power of each IoT device is 27 dBm and the minimum transmit power is 20 dBm. Unless stated, it is assumed that the system total available bandwidth B = 1.4 MHz consisting of 6 RBs. The noise power density $\sigma^{2\;}\;$= −174 dBm/Hz. The main simulation parameters are summarized in Table 2.

To verify the efficiency of the proposed scheduler; the proposed algorithm is compared with the maximum CQI scheduling algorithm. According to [35], the best CQI algorithm shows its superiority in the achieved sum rate and spectral efficiency. The idea of the best CQI is based on scheduling the *N_sel* nodes which have the maximum CQI regardless of any other parameters.

**Figure 5:**
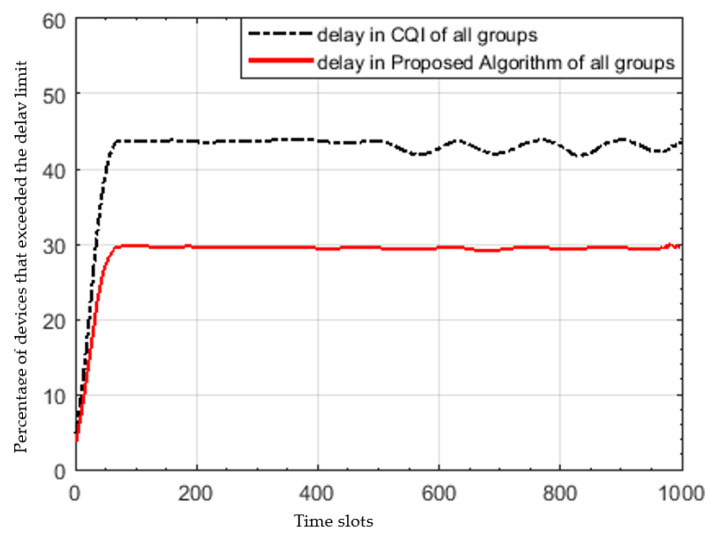
Percentage of devices exceeding the application delay limit at each time slot.

Figure 5 shows the percentage of IoT devices that exceeds the delay limit, and as clearly seen the proposed algorithm outperforms the maximum CQI and the gap increases with the time, until it reaches a certain point at which the percentage of devices exceeding the delay limit saturates in both algorithms. This is due to its receiving large requests and scheduling *N_sel* nodes only in each TTI while rejecting the rest of nodes. The percentage of devices exceeding the delay tolerance saturates in the proposed algorithm to nearly 30% but around 44% in maximum CQI. The proposed algorithm gives priority to the nodes of the maximum buffer and least delay tolerance, resulting in 31.8% delay improvement.

**Figure 6:**
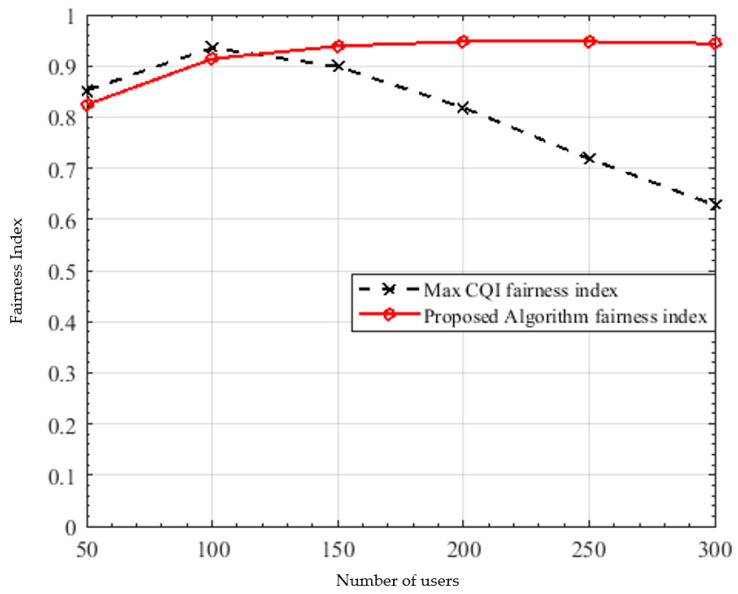
Fairness index versus number of users.

To evaluate the fairness of the proposed algorithm; the formula of Jain’s fairness index in [8] is used, which is given by

(44) $$\text{Jain’s fairness index}=\frac{{\left(\sum_{k=1}^{K}R_{k}\right)}^{2}}{K\sum_{k=1}^{K}R_{k}^{2}}$$

where $R_{k}$ represents the rate of user *k* and *K* is the total number of users. The fairness index values are confined between 0 and 1 such that the maximum value is achieved when the users have equal data rates. As obviously seen in Figure 6, the proposed algorithm is significantly fairer than the best CQI, since scheduling is performed sequentially from all groups, in addition to the fair power allocation algorithm used unlike the best CQI; resulting in 50.8% fairness enhancement at 300 users.

**Figure 7:**
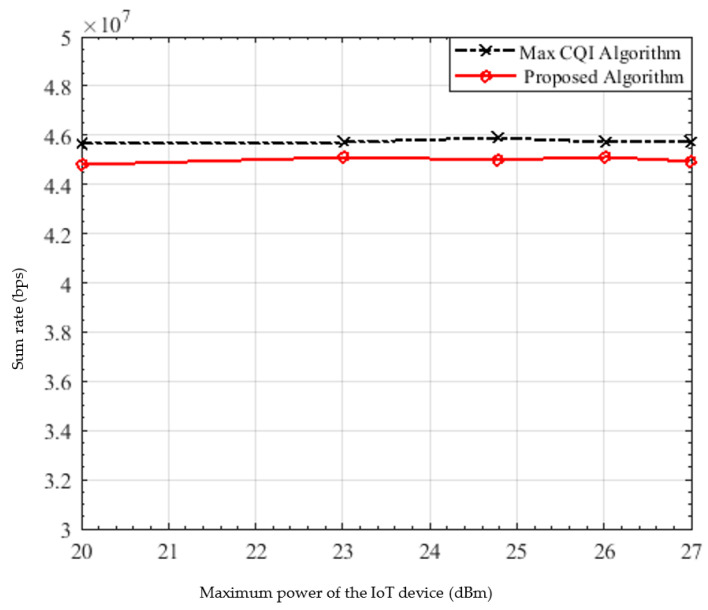
Sum rate versus the power of the IoT devices.

In Figure 7, the relationship between the transmitted power of the IoT devices and the sum rate is shown. As expected, the sum rate of the maximum CQI is greater than the proposed algorithm by only 1.6%; therefore, they still have a very close performance. By increasing the IoT device’s maximum power, the sum rate is nearly the same and does not change; this is due to the slight increase in the device’s maximum power that it does not affect the sum rate.

**Figure 8:**
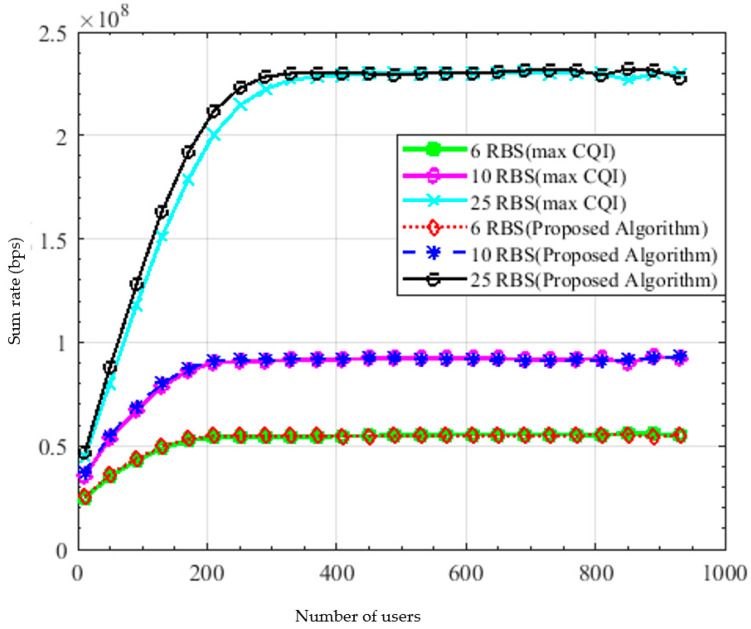
Sum rate versus number of users.

Figure 8 shows a comparison between the proposed algorithm and the best CQI in the achieved sum rate versus the number of users at different bandwidths; in case of 6, 10 and 25 resource blocks. They achieved nearly the same performance, which proves the superiority of the proposed algorithm. As the number of users increases, the sum rate increases to a certain saturation point after which the sum rate is almost constant due to scheduling the maximum number of users *N_max_* in every TTI achieving almost the same sum rate. It can be noticed that the sum rate in case of 25 RBs outperforms that in case of 6 RBs and 10 RBs, while it is the least in the case of 6 RBs. It is observed that the saturation point is shifted to the right as the number of RBs increases, meaning that as the number of RBs increases, the sum rate saturates at larger number of users.

**Figure 9:**
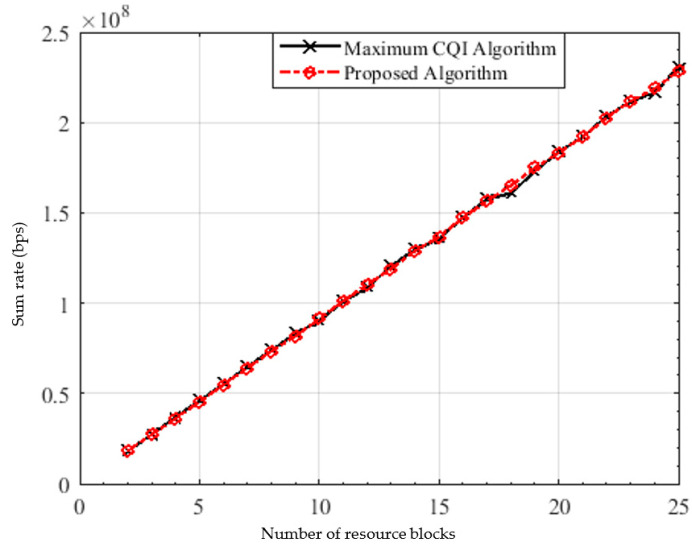
Sum rate versus the number of RBs.

Figure 9 shows the achieved sum rate versus the bandwidth (number of RBs) with maximum 2 users allocated to the resource block. The spectral efficiency of the proposed algorithm is almost closer to that of the maximum CQI, which verifies the efficiency of the proposed algorithm in terms of spectral efficiency.

The results obtained in Figure 7, Figure 8 and Figure 9, show the excellence of the proposed algorithm in terms of spectral efficiency and sum rate. This is because the proposed algorithm cannot exceed the best CQI which represents the upper bound in throughput and spectral efficiency. However, the proposed algorithm records a very close performance.

**Figure 10:**
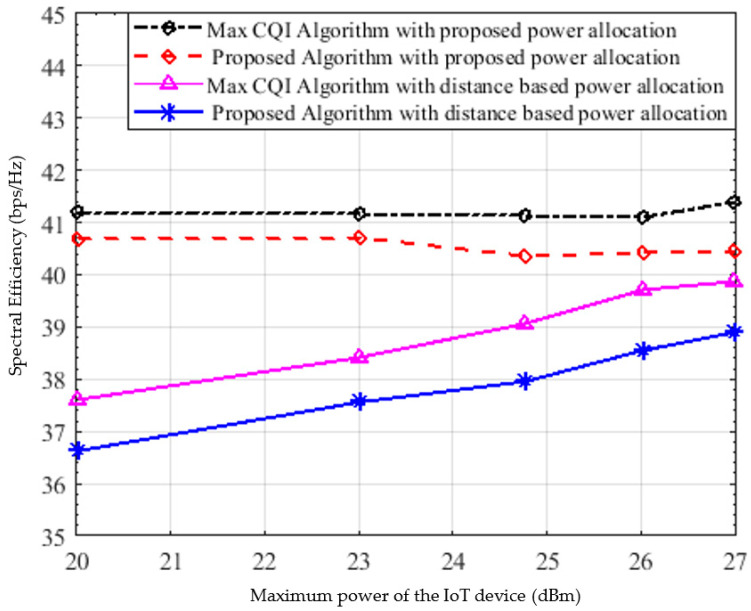
Spectral efficiency versus the power of the IoT devices.

Figure 10 shows the spectral efficiency versus the maximum power of the IoT devices comparing the performance of both the proposed resource allocation algorithm and the best CQI once using the proposed power allocation and once using distance-based power allocation. As can be clearly seen, the proposed power allocation outperforms the distance=based power allocation. Moreover, as expected, the maximum CQI is superior in terms of spectral efficiency which is on average 41.2 bps/Hz followed by the proposed algorithm which achieves very close performance, nearly 40.5 bps/Hz and about 1.69% decrease only.

**Figure 11:**
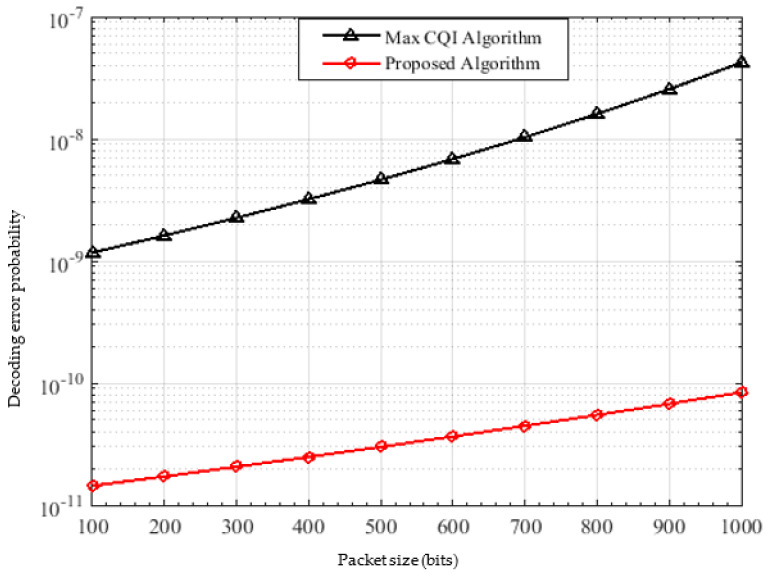
Decoding error probability versus the packet size.

To evaluate the reliability of the algorithms, the relationship between the packet size and probability of decoding error is shown in Figure 11. As expected, as the packet size increases, the probability of error increases in both algorithms. However, the probability of error ε in the proposed algorithm is significantly lower than that of the max CQI, where at packet size = 1000 bits, ε is about $8.5*10^{-11}$ and $4.2*10^{-8}$ in the proposed algorithm and the max CQI respectively, resulting in nearly 99.7% performance improvement.

The computational complexity of both the proposed algorithm and the best CQI is $O\left(KN\right)$ which means that they have linear complexity in the number of users and number of RBs. However, in [36], resource allocation for uplink multi carrier NOMA is developed using graph theory with complexity $\;O\left(KN^{3}\right)$. The shortest processing time (SPT) strategy is presented in [37] for uplink NOMA with complexity $\;O\left(N^{2}\right)$. Thus, the proposed algorithm has a worthy complexity improvement.

## 7. Conclusions

In this paper, an uplink NOMA resource allocation algorithm is proposed for a UAV-IoT-based communication network serving a large number of IoT devices of different applications. The optimization problem is formulated under constraints with the objective to maximize the data rate and minimize the delay. The scheduler works in time domain to optimize the delay, and in frequency domain to optimize the data rate. The power allocation is used to ensure fair data rates between users allocated the same RB. The simulation results show that the proposed algorithm significantly enhances the system fairness, delay and reliability, in addition to achieving a spectral efficiency and sum rate that are nearly closer to the system upper bound.

## Figures and Tables

**Table 1:** Values of optimal elevation angle of each environment.

Environment	$\theta_{optimum}$
Suburban	20.34°
Urban	42.44°
Dense urban	54.62°
High-rise urban	75.52°

**Table 2:** Simulation parameters.

Symbol	Description	Value
a	Environment Constant	4.88
b	Environment Constant	0.43
$\eta_{LOS}$	Line of sight Environment Constant	0.1
$\eta_{NLOS}$	Non-Line of sight Environment Constant	21
$\theta_{optimum}$	Optimal elevation angle	20.34°
$\sigma^{2}$	Noise power density	−174
U	Number of UAVs	1
$P_{min}$	IoT device minimum power	100 mW–20 dBm
$P_{max}$	IoT device maximum power	500 mW–27 dBm
$R_{c}$	Radius of the cell	1 km
TTI	Time slot	1 ms
	Simulation time	1 s
$D_{limit}$	Maximum delay limit	{10,20,30,40} ms
λ	Arrival rate per group	{100,250,600,400} (packets/s)
N	Total number of devices	300
*D*	Packet size	100 bits
*m*	Channel blocklength	100 symbols

## Data Availability

Not applicable.
